# A multi-subband Monte Carlo study on dominance of scattering mechanisms over carrier transport in sub-10-nm Si nanowire FETs

**DOI:** 10.1186/s11671-016-1249-4

**Published:** 2016-01-27

**Authors:** Hoon Ryu

**Affiliations:** National Institute of Supercomputing and Networking, Korea Institute of Science and Technology Information, Daejeon, 305-806 Republic of Korea

**Keywords:** Si Nanowire, Scattering dominance, Hole mobility, Multi-subband Monte Carlo simulations, Schrödinger-Poisson, **PACS Codes:**, 73.21.Hb, 73.22.-f, 73.50.Dn

## Abstract

Dominance of various scattering mechanisms in determination of the carrier mobility is examined for silicon (Si) nanowires of sub-10-nm cross-sections. With a focus on *p*-type channels, the steady-state hole mobility is studied with multi-subband Monte Carlo simulations to consider quantum effects in nanoscale channels. Electronic structures of gate-all-around nanowires are described with a 6-band *k* · *p* model. Channel bandstructures and electrostatics under gate biases are determined self-consistently with Schrödinger-Poisson simulations. Modeling results not only indicate that the hole mobility is severely degraded as channels have smaller cross-sections and are inverted more strongly but also confirm that the surface roughness scattering degrades the mobility more severely than the phonon scattering does. The surface roughness scattering affects carrier transport more strongly in narrower channels, showing ∼90 % dominance in determination of the mobility. At the same channel population, [110] channels suffer from the surface roughness scattering more severely than [100] channels do, due to the stronger corner effect and larger population of carriers residing near channel surfaces. With a sound theoretical framework coupled to the spatial distribution of channel carriers, this work may present a useful guideline for understanding hole transport in ultra-narrow Si nanowires.

## Background

Semiconductor devices have been continuously downscaled ever since the invention of the first transistor [1], leading the size of a single building block of modern devices to a few nanometers (nm). Silicon (Si) nanowires have gained attention as advanced field effect transistors (FETs) due to their enhanced control over channel electrostatics [2, 3] and as one of strong candidates for ultra-narrow interconnects [4, 5]. Solid understanding of such nanoscale devices is critical as it can accelerate potential device designs and should be always based on quantum physics since their material and electrical properties are strongly dependent on channel dispersions and quantization of subband energies [2–6].

Scattering-induced suppression of channel conductance in ultra-narrow nanowire FETs has been a particular interest to researchers, invoking theoretical studies based on Non-Equilibrium Green’s Function (NEGF) approach [7–10]. They however only focused on the scattering induced by either phonons or surface roughness due to extremely large computing loads [7–10]. Scattering-involved carrier transport in nanostructures has been also explored with multi-subband Monte Carlo (MSMC) simulations coupled to Boltzmann transport theory [11–19], which can include multiple scattering mechanisms with reasonable computing loads. Most of previous works however focused on studying electron transport [11–16]. While hole transport in nanostructures has been studied [17–19], results were limited by only including the phonon scattering under no external biases [17] and only focused on the mobility in 1D-confined inversion layers or double-gated structures rather than free-standing gate-all-around nanowires [18, 19].

This work presents a comprehensive theoretical study on suppression of carrier transport in extremely narrow, gate-all-around (GAA) *p*-type Si nanowires of sub-10-nm cross-sections. Scattering-induced degradation of the low-field hole mobility under channel modulation has been rigorously investigated with Schrödinger-Poisson and MSMC simulations. Dependency of the hole mobility on carrier densities, transport directions, and cross-section sizes of nanowire channels has been understood. Dominance of scattering mechanisms in determination of the carrier mobility has been analyzed to find the major scattering mechanism that suppresses the mobility in sub-10-nm channels. Presenting a sound theoretical framework for studying the effect of individual scattering mechanism on the mobility that has been rarely discussed based on the spatial distribution of channel carriers, this work may serve as a useful guideline for understanding hole transport in sub-10-nm ultra-narrow Si nanowires.

## Methods

### Electronic Structure

**Fig. 1:**
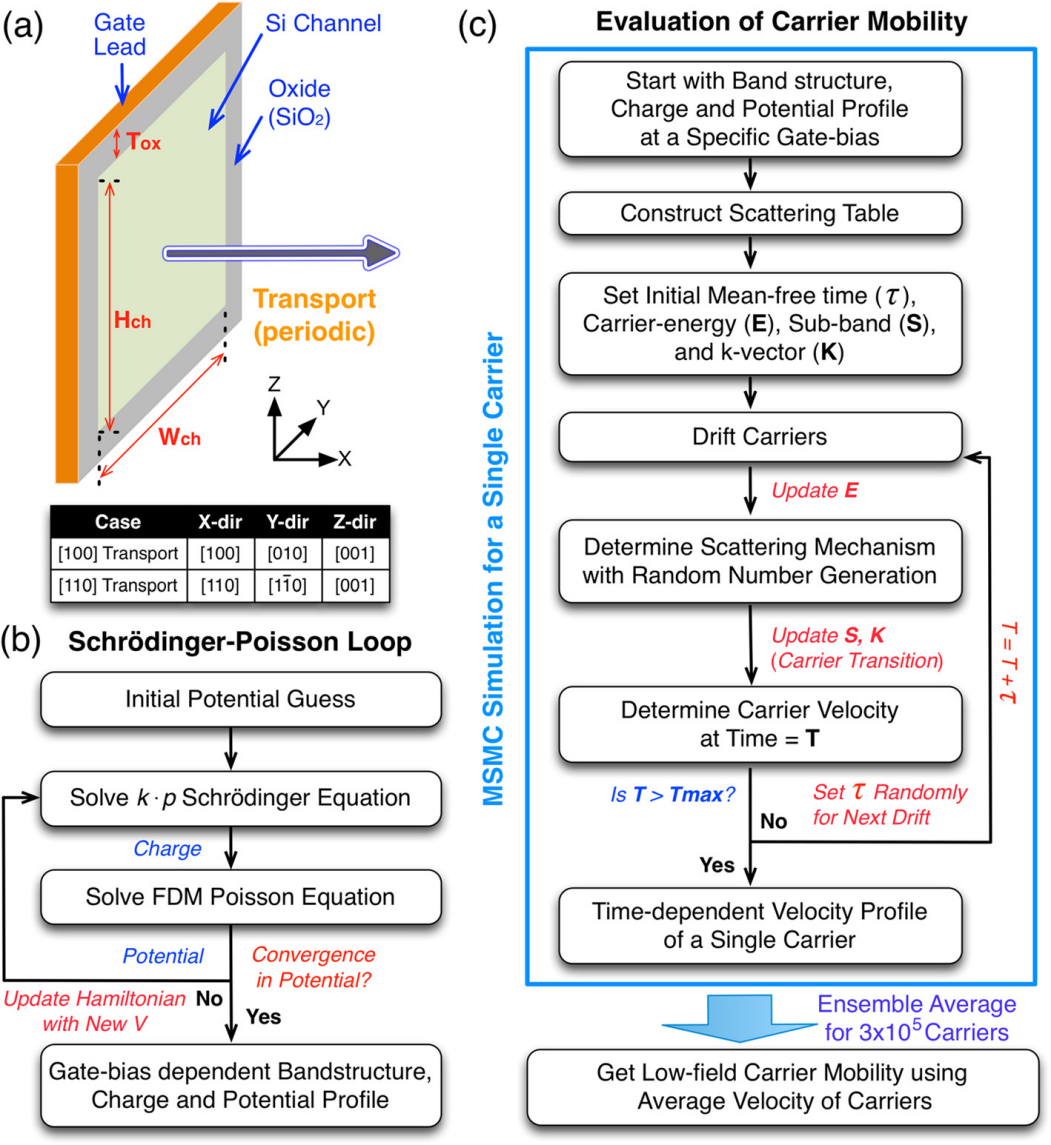
Approach to modeling of Si nanowires. **a** The geometry of gate-all-around Si nanowires. Long channels are represented by supercells with a periodic boundary condition along transport directions. 1.0-nm-thick oxide layer is assumed for all the simulated nanowires. **b** The process of Schrödinger-Poisson simulations. A 2D *k* · *p* Schrödinger and a 2D Poisson equation are solved self-consistently to get bandstructures and electrostatics under gate biases. **c** The process for evaluation of the carrier mobility. The velocity of a single carrier is calculated by solving a 1D Boltzmann transport equation with Monte Carlo approach, including scattering induced by the acoustic/non-polar optical phonons and channel surface roughness. The carrier mobility is then evaluated with the velocity that is ensemble-averaged for 3 × 10^5^ carriers

Figure 1a gives a conceptual illustration of how Si nanowires are described in this work. Channels are assumed to be long and have rectangular cross-sections, thus being represented by supercells with a periodic boundary condition along transport directions. Free-standing nanowire channels are assumed to be intrinsic, trap-free, and perfectly surrounded by gate electrodes (GAA structure). Electronic structures of nanowire channels are described using a 6-band *k* · *p* model [20], with a focus on valence bandstructures to study hole transport. All the channels simulated in this work are assumed to have 1.0-nm-thick oxide layer regardless of transport directions and cross-section sizes.

### Electrostatics and Mobility

Channel bandstructures and electrostatics (charge and potential profiles) under gate biases are determined self-consistently with Schrödinger-Poisson simulations. Figure 1b shows the process of our Schrödinger-Poisson loop, where a *k* · *p* Schrödinger solver runs coupled to a Poisson solver that discretizes simulation domains with finite difference method, until the potential profile converges within a criterion defined by users. Hole wavefunctions are not allowed to penetrate into the oxide layer, and therefore the charge density is assumed to be always zero inside the gate insulator for calculation of the channel potential. We note that all the simulations in this work are performed with in-house solvers, using a mean square error of 10^−5^ eV as a convergence criterion.

Once channel electrostatics and bandstructures are obtained from Schrödinger-Poisson simulations, the velocity of a single hole carrier is determined by solving the 1D Boltzmann transport equation with MSMC approach, where we assume that carrier transport happens under an electric field of 10^5^ V/m, and is affected by scatterings induced by acoustic phonons, non-polar optical phonons, and channel surface roughness. The phonon and surface roughness scattering rate are calculated with the numerical model and parameters used by Ramayya et al. [12] and Michielis et al. [19], respectively, where we extracted effective masses and nonparabolicity factors of the highest 45 subbands from *k* · *p* dispersions that are obtained self-consistently, and only considered wavefunctions at *Γ*-point (*k*=0) to compute hole-scattering form factors as previous studies did [17–19]. While carrier transport can be significantly affected by the impurity coulomb scattering if nanowires have a non-negligible density of channel impurities or traps, this work ignores the effect of the impurity scattering assuming all the channels are intrinsic and free from traps, as done by previous studies [12, 13, 15]. The carrier mobility is then evaluated by taking the (time) ensemble average of velocity profiles of 3 × 10^5^ carriers. Figure 1c shows the overall process of simulations in detail, including the process of MSMC simulations for evaluation of the velocity of a single carrier. We note that the numerical model for the surface scattering rate employed in this work only considers surface roughness along transport directions, ignoring correlation among channel surfaces for evaluation of the scattering rate. Our results therefore may be further improved with more sophisticated models [21, 22], which consider surface roughness along both transport and confinement directions without necessarily assuming decoupled surface planes.

### Simulation Setup

[100] and [110] transport-oriented GAA nanowire channels are considered assuming a room temperature and a zero flat band voltage. For each transport direction, six cross-section sizes (3, 4, 5, 6, 8, and 10 nm) are simulated under gate voltages ranging from 0 to −1 V with a step of −0.1 V. Our in-house Schrödinger-Poisson and MSMC solver run in parallel and complete the simulations in about 1 ∼4 h with 128 Intel Xeon x5570 CPUs depending on gate biases and channel sizes.

### Domainance of Scattering Mechanisms

Recently, Niquet et al. have introduced a paradigm for definition of the mobility driven by a single scattering mechanism, which uses the phonon scattering (PH) as a reference to extract the mobility driven by the surface roughness scattering (SRS), satisfying Matthiessen’s rule in the quantum regime [23]. Following this paradigm, we can evaluate dominance of PH and SRS in determination of the hole mobility. With Matthiessen’s rule, the total scattering probability can be expressed as a sum of individual scattering probabilities. Since a scattering probability is inversely proportional to the mean-free time [24], the total scattering probability of a single hole carrier in nanowire channels (*P*_SCAT_) becomes as shown in Eq. 1:

(1) $$ P_{\text{SCAT}} \simeq \frac{1}{\tau} = \frac{1}{\tau_{\text{PH}}} + \frac{1}{\tau_{\text{SRS}}},  $$

where *τ* is the total mean-free time of a single carrier; *τ*_PH_ and *τ*_SRS_ are the mean-free time due to PH and SRS, respectively. Dominance of PH and SRS in determination of the hole mobility (*D*_PH_,*D*_SRS_) can be then quantified by the probability that scattering is induced by either phonons or channel surface roughness, given that a carrier experiences scattering. This *conditionalprobability* can be calculated using Eqs. 2 and 3:

(2) $$\begin{array}{@{}rcl@{}} D_{\text{PH}} &=& \frac{P_{\text{PH}}}{P_{\text{SCAT}}} = \frac{\tau_{\text{SRS}}}{\tau_{\text{PH}}+\tau_{\text{SRS}}}\\ &=& \frac{\mu_{\text{SRS}}}{\mu_{\text{PH}}+\mu_{\text{SRS}}} (\because\tau\propto\mu), \end{array} $$

(3) $$\begin{array}{@{}rcl@{}} D_{\text{SRS}} &=& \frac{P_{\text{SRS}}}{P_{\text{SCAT}}} = \frac{\tau_{\text{PH}}}{\tau_{\text{PH}}+\tau_{\text{SRS}}}\\ &=& \frac{\mu_{\text{PH}}}{\mu_{\text{PH}}+\mu_{\text{SRS}}} (\because \tau \propto \mu),  \end{array} $$

where *P*_(PH,SRS)_ is the probability of scattering induced by phonons and surface roughness, respectively; *μ*_(PH,SRS)_ is the mobility driven by the corresponding scattering.

## Results and Discussion

### Mobility of Hole Carriers

**Fig. 2:**
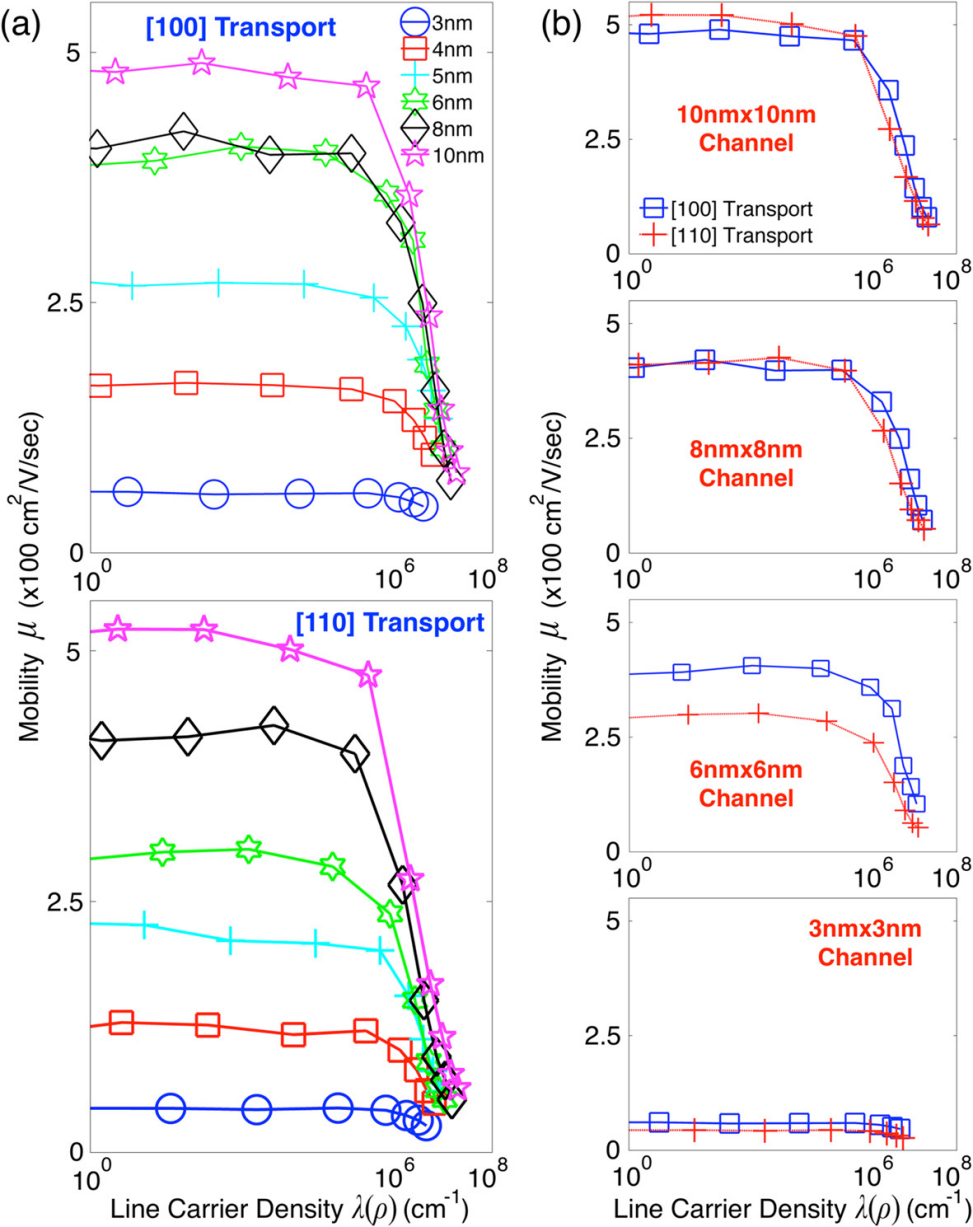
Steady-state mobility of hole carriers. **a** Sensitivity of the hole mobility to cross-section sizes and channel population. The mobility is generally degraded as channels become narrower. The mobility is also degraded as channels are inverted more strongly, and the degradation becomes particularly rapid when the density of inversion carriers (*λ*(*ρ*)) exceeds ∼10^6^cm^−1^. **b** Variation of the mobility at selected cross-section sizes. At cross-sections ≤ 6 nm × 6 nm, the hole mobility becomes lower in [110] channels than [100] ones. The same pattern is also observed at larger cross-sections if channels are in the regime of strong inversion (*λ*(*ρ*)>∼10^6^ cm^−1^)

Low-field characteristics of the hole mobility in sub-10-nm nanowires are summarized in Fig. 2a, where we plotted the steady-state carrier mobility as a function of line densities of inversion carriers (*λ*(*ρ*)) instead of gate voltages for convenience in discussion. Here, we observe that variation of the mobility strongly depends on both channel cross-section sizes and carrier densities. In general, the mobility suffers from severe degradation as nanowires become narrower, although the degradation is not remarkable for some cases—for example, in [100] nanowires, the mobility in a 6 nm × 6 nm channel does not show a clear reduction compared to the 8 nm × 8 nm case. The mobility is also degraded as channels are inverted more strongly, and the degradation becomes rapid if the inversion is strong enough to fill hole carriers with *λ*(*ρ*)>∼10^6^ cm^−1^. We note that the pattern observed in Fig. 2a are generally reasonable since stronger structural confinement stemming from narrower channels increases the hole-phonon scattering rate [17, 25], and the chance for scattering induced by surface roughness would also increase in more populated and narrower channels. As channels become broader, the mobility eventually reaches ∼500 cm^2^/V ·s, the value in low-doped bulk Si [26], regardless of transport directions.

Simulation results also indicate that behaviors of the mobility depend on transport directions. To describe this dependency more clearly, we depicted the mobility in [100] and [110] channels in Fig. 2b with a set of subplots, where each subplot shows the result at a specific cross-section size. When channels have cross-sections ≥ 8 nm × 8 nm, the mobility experiences severer degradation in [110] channels than [100] ones only if channels are inverted strongly (*λ*(*ρ*)>∼10^6^ cm^−1^). In narrower channels, however, the mobility always becomes lower in [110] channels than [100] ones regardless of inversion carrier densities, where the gap between [110] and [100] mobility becomes particularly large in a 6 nm × 6 nm channel.

**Fig. 3:**
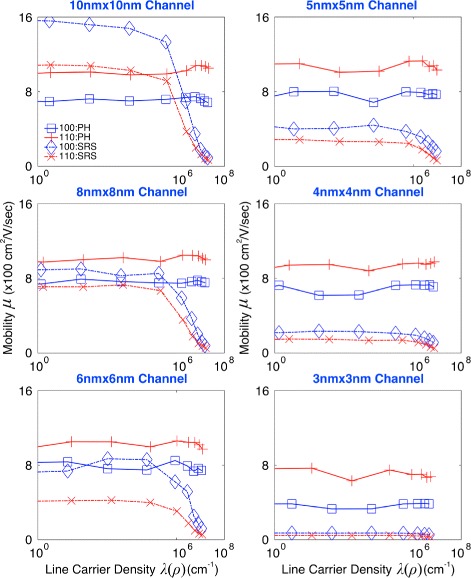
Mobility driven by phonon and surface roughness scattering. The mobility driven by the phonon scattering (PH) becomes higher in [110] nanowires than [100] ones, while the mobility driven by the surface roughness scattering (SRS) generally shows an opposite behavior. Although the PH-driven mobility does not show a clear sensitivity to channel population, the SRS-driven mobility is severely degraded as channels are inverted more strongly. The SRS-driven mobility also generally decreases as channels become narrower and eventually drops below the PH-driven mobility regardless of transport directions and channel population at cross-sections ≤ 5 nm × 5 nm

To investigate the abovementioned behaviors more rigorously, we computed the hole mobility including PH only and extracted the SRS-driven mobility using the method presented in [23]. Figure 3 summarizes the results, showing behaviors of the PH- and SRS-driven mobility in [100] and [110] nanowires. The first remarkable point we observe is that the PH-driven mobility generally becomes higher in [110] than [100] nanowires, where the reason can be found from the higher injection velocity of hole carriers in [110] Si channels [27]. The SRS-driven mobility, however, shows an opposite pattern and therefore generally becomes lower in [110] channels than [100] ones. While the PH-driven mobility does not show a clear sensitivity to carrier densities, the SRS-driven mobility clearly decreases with increasing channel population, exhibiting a pattern similar to what is observed from behaviors of the total mobility (Fig. 2a). The SRS-driven mobility also generally decreases as channels become narrower. This pattern becomes more remarkable in [110] than [100] channels and explains the lager reduction of the total mobility in [110] channels, particularly when the cross-section is reduced from 8 nm × 8 nm to 6 nm × 6 nm. While the PH-driven mobility does not exhibit a clear discrepancy in both [110] and [100] channels when the channel size is reduced from 8 nm × 8 nm to 6 nm × 6 nm (Fig. 3), the degradation of the SRS-driven mobility is much severer in [110] channels, causing more remarkable reduction of the total mobility than in [100] channels. We note that the SRS-driven eventually drops below the PH-driven mobility regardless of transport directions and carrier densities when channels have cross-sections ≤ 5 nm × 5 nm.

So far, we have discussed low-field behaviors of the hole mobility in sub-10-nm GAA Si nanowires with particular interest in their dependency on cross-section sizes, transport directions, and inversion carrier densities. However, results shown in Figs. 2 and 3 raise a question that has yet to be clearly answered in a theoretical perspective: why hole carriers generally experience severer degradation of the total and SRS-driven mobility in [110] channels than [100] ones? Reasons of this question will be pursued in further detail in the next subsection by understanding the dominance of scattering mechanisms in determination of the hole mobility.

### Dominance of Scattering Mechanisms

**Fig. 4:**
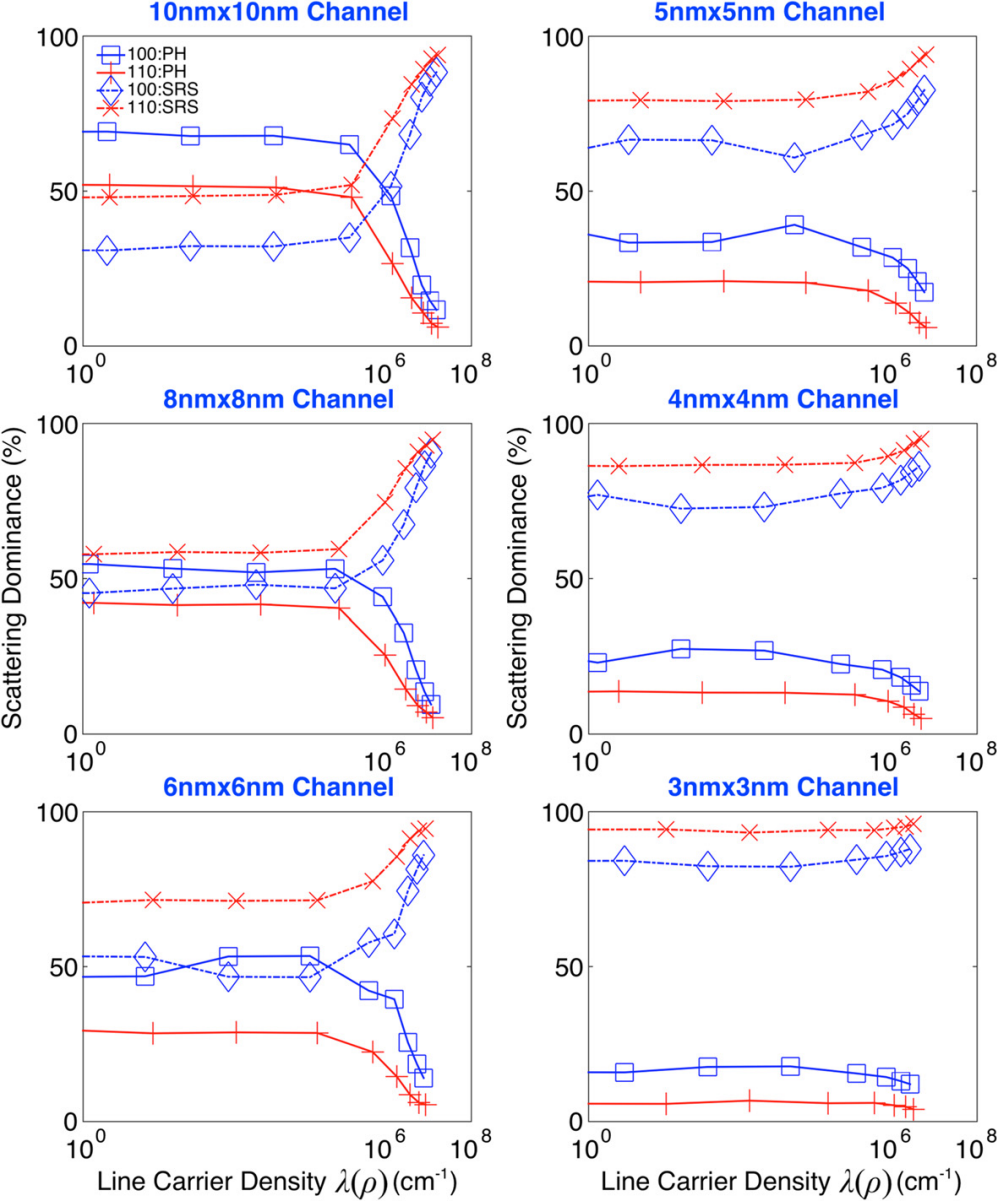
Dominance of scattering mechanisms over hole mobility. Degradation of the hole mobility in sub-10-nm channels turns out to be dominated by the surface roughness scattering (SRS). In 10 nm × 10 nm channels, the dominance of SRS in determination of the mobility becomes larger than 50 % exceeding that of the phonon scattering (PH) when channels are strongly inverted with a line density of carriers (*λ*(*ρ*))>∼10^6^ cm^−1^. The dominance of SRS generally increases as channels become narrower, and the increase becomes more rapid in [110] channels. Consequently, in [110] channels, SRS dominates PH regardless of carrier densities at cross-sections ≤ 8 nm × 8 nm, while the same pattern is observed in [100] channels at cross-sections ≤ 5 nm × 5 nm

To find major scattering mechanisms for degradation of the hole mobility in sub-10-nm Si nanowires, we quantified the dominance of PH and SRS in determination of the total hole mobility using Eqs. (2) and (3) and the PH-/SRS-driven mobility shown in Fig. 3. Results are summarized in Fig. 4, where we observe a general pattern that SRS affects the total hole mobility more strongly than PH does. When nanowires have a 10 nm × 10 nm cross-section, the dominance of SRS starts to exceed that of PH, becoming larger than 50 % in the regime of strong inversion (*λ*(*ρ*)>∼10^6^ cm^−1^). The dominance of SRS increases in general as channels become narrower, and the increase becomes more rapid in [110] channels. In [110] channels, therefore, SRS dominates PH in determination of the total mobility regardless of carrier densities at cross-sections ≤ 8 nm × 8 nm, while the same pattern is observed in [100] channels at cross-sections ≤ 5 nm × 5 nm. At a 3 nm × 3 nm cross-section, the average dominance of SRS becomes ∼94 % in [110] channels and ∼84 % in [100] ones.

**Fig. 5:**
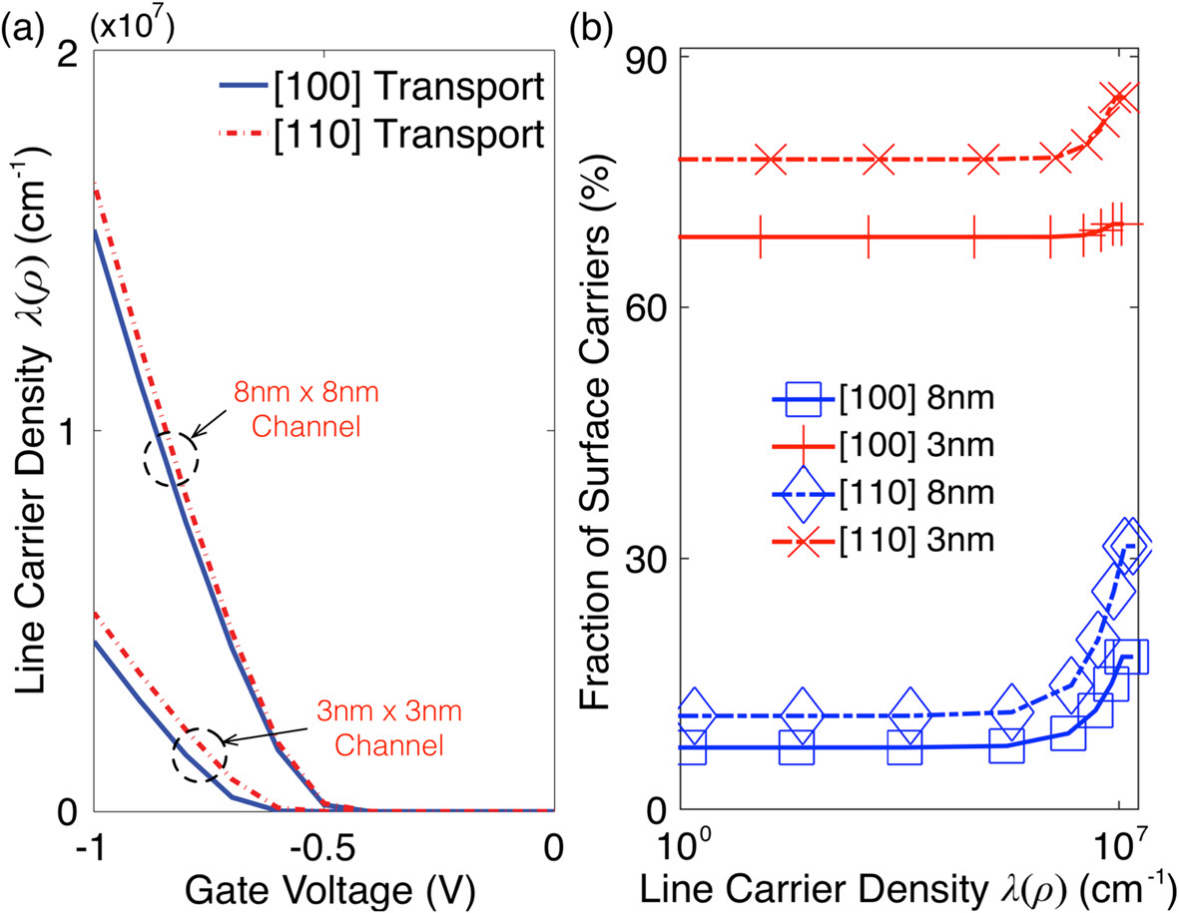
Carrier density and ratio of surface carrier population to the total one. **a** The line density of hole carriers that is plotted as a function of gate biases for a 3 nm × 3 nm and a 8 nm × 8 nm cross-section. A negatively increasing gate voltage not only fills more carriers but generally creates stronger band bending in channels, increasing the chance for carriers to experience the corner effect. **b** The ratio of surface carrier (staying within 1.0 nm from channel surfaces) population to the total one that is plotted as a function of line densities of carriers for a 3 nm × 3 nm and a 8 nm × 8 nm cross-section. Although the corner effect may not be remarkable in narrower channels where cross-sections are not large enough to have clear band bending, the chance for the surface roughness scattering would be still high since more carriers are then placed near channel surfaces

Figure 5a shows the line density of inversion carriers in [100] and [110] channels as a function of gate voltages. A negatively increasing gate voltage fills more carriers and creates stronger band bending in channels. Generally, stronger band bending would not only fill more carriers near channel surfaces but also cause the stronger corner effect in rectangular channels [28], increasing the probability that a single hole carrier experiences SRS. The corner effect may not be remarkable in narrower channels, where cross-sections would not be large enough to have clear band bending. But the chance for SRS would still become higher with increasing population of carriers residing near channel surfaces. Figure 5b shows the ratio of surface carrier population to total one, as a function of line densities of carriers, where *surfacecarriers* are defined as the ones staying within 1.0 nm from channel surfaces. Here, we observe that the ratio increases as channels are inverted more strongly, and narrower channels have more surface carriers at the same channel population. Since [110] channels show larger population of carriers at the same gate voltage and place more carriers near surfaces at the same channel population, we expect that a single hole carrier in [110] channels would have higher possibility to experience SRS than in [100] channels.

**Fig. 6:**
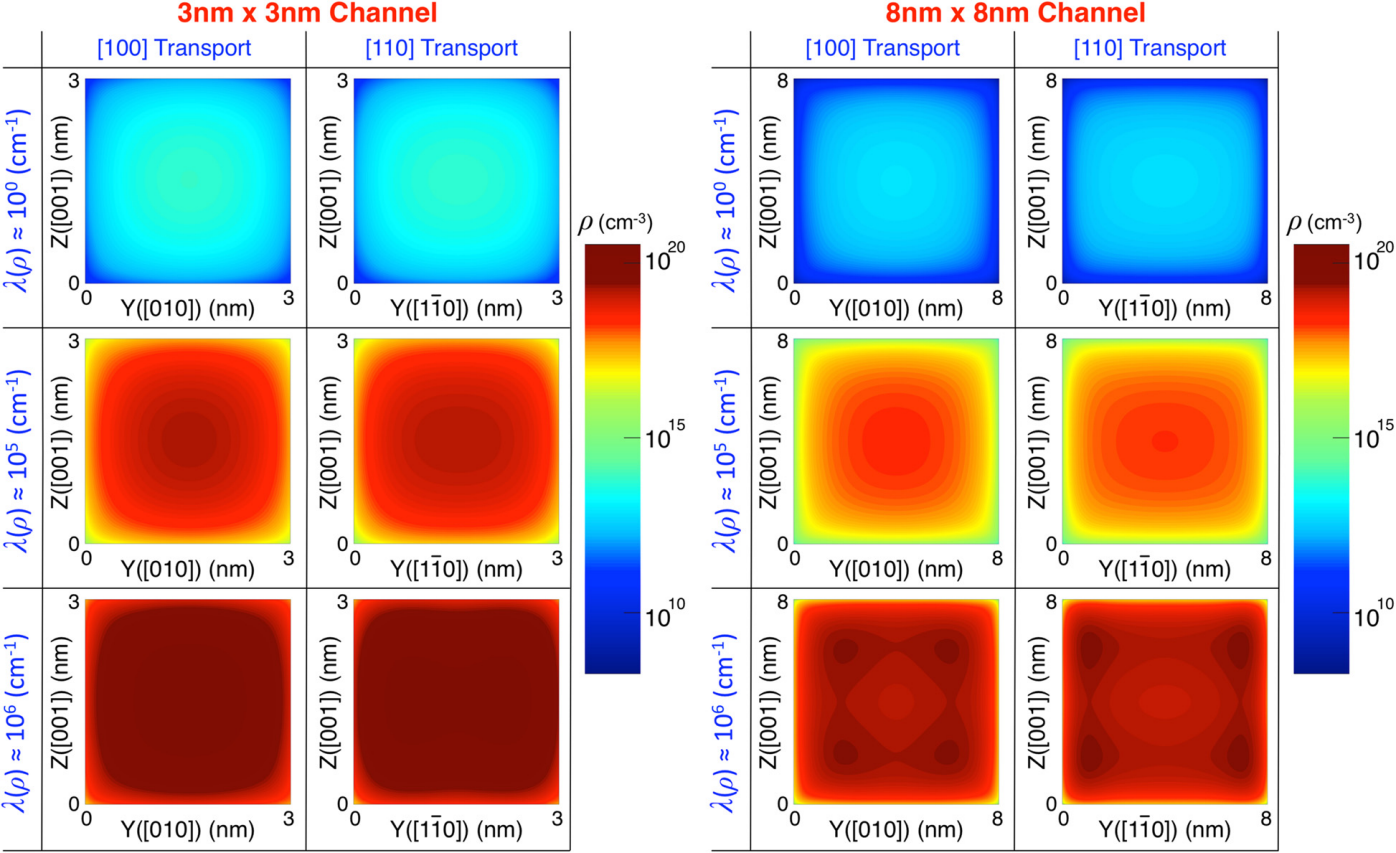
Carrier volume density on channel cross-section. The volume density of hole carriers is plotted on a 3 nm × 3 nm (*left*) and a 8 nm × 8 nm cross-section (*right*) as a function of channel population. In general, [110] channels have broader distributions of carriers than [100] channels at the same population. Results of the 8 nm × 8 nm case clearly show that the corner effect also becomes stronger in [110] than [100] channels when channels are under strong inversion and broad enough to have clear band bending

To understand the correlation between the corner effect and transport directions more clearly, we plotted the carrier distribution on a 3 nm × 3 nm and a 8 nm × 8 nm cross-section in Fig. 6, as a function of channel population. Here, we observe that the distribution generally becomes broader in [110] than [100] channels at the same population, confirming again that [110] channels have more surface carriers than [100] channels as shown in Fig. 5b. As indicated by the 8 nm × 8 nm case in Fig. 6, the corner effect would also become stronger in [110] than [100] channels if channels are under strong inversion and broad enough to have clear band bending. The two factors discussed in Figs. 5b and 6, i.e., the larger population of surface carriers and stronger corner effect, explain well why [110] channels suffer from SRS more severely and thus have a larger dominance of SRS than [100] channels do, presenting the answer to the question raised in the previous subsection. While the latest models for SRS [21, 22] may cause quantitative changes in dominance of individual scattering mechanism (Fig. 4), the main message of this subsection would not lose its validity, being supported by the abovementioned two factors that are driven by the spatial distribution of channel carriers.

It should be noted that previous theory works have also studied the hole mobility in ultra-narrow Si nanowires by solving the linearized Boltzmann transport equation coupled to the atomistic tight-binding approach [29, 30]. In particular, Neophytou et al. [30] have simulated free-standing GAA Si nanowires and have demonstrated that the mobility becomes higher in [110] than [100] nanowires and increases in [110] nanowires as channels become narrower, establishing a qualitative connection to the similar experimental study [31]. While [30] presents good theoretical background for understanding behaviors of the hole mobility in GAA Si nanowires with results contradictory to ours, we claim that it may have underestimated the effect of SRS in [110] nanowires, since the increase of the hole mobility predicted by modeling is much larger than what has been observed experimentally [31]. Moreover, another recent experimental study done by Nomura et al. [32] has even shown that the hole mobility generally suffers from severe degradation in narrower [110] channels, presenting a qualitative connection to our results and a sound evidence for underestimation of the effect of SRS in [30].

## Conclusions

We have presented a comprehensive theoretical study on the degradation of the hole mobility in ultra-narrow sub-10-nm silicon (Si) nanowires. Gate-all-round (GAA) long channels are represented by supercells with a periodic boundary condition along transport directions. Bias-dependent channel bandstructures and electrostatics are determined self-consistently by solving a 6-band *k* · *p* Schrödinger equation with a Poisson equation. The low-field hole mobility is evaluated by solving a 1D Boltzmann transport equation with Monte Carlo simulations. Dominance of the phonon scattering (PH) and the surface roughness scattering (SRS) in determination of the total hole mobility is quantified with Matthiessen’s rule, where the PH-driven mobility is used as a reference to extract the SRS-driven mobility.

Here, we observe a general pattern that the mobility is severely degraded as channels become narrower and more populated and confirm SRS as the major scattering mechanism for the mobility degradation by examining its dominance in sub-10-nm narrow channels. Although [110] channels show higher PH-driven mobility than [100] channels do, the total mobility becomes lower in [110] channels due to the SRS-driven mobility. The reason why SRS has larger dominance in [110] channels than [100] ones is also understood by investigating the spatial distribution of channel carriers and the population of carriers residing near channel surfaces. With solid discussion for the effect of individual scattering mechanism on the hole mobility, this work expands the scope of understanding hole transport to free-standing sub-10-nm GAA Si nanowires, which has been rarely covered experimentally.
